# Vocation or Profession?

**Published:** 1919-01-25

**Authors:** 


### THE MATRONS' AND SISTERS' DEPARTMENT.

### VOCATION OR PROFESSION?

Only since the war broke out has the nursing profession attracted to itself anything like appreciable attention. Before, a nurse's life was a closed book to the majority of people, and even yet to many the life of the nurse has no interest at all.

In the minds of some the nurse took rank with a religieuse, and we are not sure that that was not the greater honour. In the minds of others the nurse ranked alongside of domestic workers, housemaids, nursemaids, and so on *ad lib.* Any old thing was good enough for a nurse—that was because they did not understand. Nurses themselves did not bother much about changing the opinions of outsiders. No writer has written a decent book about nurses or the nursing profession; any book that has been written up to now presents nurses as sordid, jealous, sentimental creatures, lacking in brain and mental capacity—in fact, the whole presentation of nursing life is sinister and sordid. And nobody takes up the cudgels in our defence. Let it be so, then; it is up to nurses themselves to crush and kill the stupid fallacies that grow and exist as facts amongst certain people.

Because a few women lower the standard of dignity and womanhood is no reason why the whole profession should be branded with infamy. Not long ago in The Nursing Mirror there appeared an article about commandants and trained nurses. All along that way of working has been a sorry muddle, but in nine cases out of ten the trouble arose over the commandant thinking the trained nurse was too familiar with the men. Scarce a trained nurse who does not corroborate that sentence.

What was at the root of this stupid bitterness? The soldiers preferred the trained nurse every time. And why should not they? Surely if there is one class of woman in the world who understands all sorts and conditions of men the trained nurse takes first place. We have watched with them through long hours of weariness and pain, it was ours to still their agony and help them to pass through the hour of terror, our privilege to give the cool courage as well as heal the body.

Trained nurses take first place with the soldiers because they understand. They understand the discipline which is always galling to young and restless natures; they had done their training in civil hospitals, and had a dignity all their own. They could not lend big houses for hospitals, or give great sums of money for the Tommies' use; but they gave themselves, cheerily, willingly, wholeheartedly, at home or abroad, ungrudging even when it came to yielding up their lives.

Nothing was too much to do for the soldiers, and to the trained nurse in many cases this work meant sacrifice. (The vocation and the profession are hopelessly mixed.—Ierne!) And now all the talk of the nurses' halo. There is not much of the halo about some of us, we are a little bit afraid, but we are very happy-hearted, and happiness goes a long way. But, seriously, a woman may be tender-hearted and true, and gentle and full of love for her profession, feeling it as a vocation pulling at her heartstrings; and yet away in the background or in the dim future she may see haunting spectres of want and worry. A nurse may feel she has a vocation, and yet at the same time know she has a mother or a helpless brother depending on her.

All the vocation in the world will not give them daily bread or free the loved ones from want or worry. To pay the price of her vocation she must see her family suffer. Then they are paying the price, and not she herself. Is she going to renounce her vocation and enter a profession where more money will be forthcoming, or is she going to be sensible and turn all her energies to making her vocation also a paying profession? We cannot live on air, though it may be possible for a time to live on love. We do not altogether want to "hang up our halo" for good, but we do want to be sensible and pull together for that oasis which is waiting for those who have the courage to go on. There is little use in becoming faint-hearted and disappointed. With the College to back us we can go far without fear of being left in the lurch. The antagonists of the College are still very bitter. That is a pity. But now is not the time for bitterness or regret. We must concentrate our energies to make our profession rank in the front row. Soon military and Red Cross hospitals will be but dreams of the past, and the great civil nursing world will come into its own again. Opportunity is knocking at our doors; are we going to open and let her in?

